# Piloting a classification framework for the types of evidence used in alcohol policymaking

**DOI:** 10.1111/dar.13599

**Published:** 2023-01-25

**Authors:** Michala Kowalski, Claire Wilkinson, Michael Livingston, Alison Ritter

**Affiliations:** ^1^ Drug Policy Modelling Program, Social Policy Research Centre UNSW Sydney Sydney Australia; ^2^ Centre for Alcohol Policy Research La Trobe University Melbourne Australia; ^3^ National Drug Research Institute and enAble Institute, Faculty of Health Sciences Curtin University Perth Australia

**Keywords:** Australia, content analysis, evidence utilisation, influence, practice knowledge

## Abstract

**Introduction:**

Most studies of alcohol policy have focussed on the role of industry. However, little is known about the evidence base used in alcohol policymaking or policymakers' actions in the field. Here, we mapped the different evidence types used in a case study to construct a classification framework of the evidence types used in alcohol policymaking.

**Methods:**

Using a case study from the state‐level in Australia, we used content analysis to delineate the evidence types cited across six phases of a policymaking process. We then grouped these types into a higher‐level classification framework. We used descriptive statistics to study how the different evidence types were used in the policymaking process.

**Results:**

Thirty‐one evidence types were identified in the case study, across four classes of knowledge: person knowledge, shared knowledge, studied knowledge and practice knowledge. The participating public preferenced studied knowledge. Policymakers preferenced practice knowledge over all other types of knowledge.

**Discussion and Conclusion:**

The classification framework expands on models of evidence and knowledge used across public health, by mapping new evidence types and proposing an inductive method of classification. The policymakers' preferences found here are in line with theories regarding the alcohol industry's influence on policymaking. The classification framework piloted here can provide a useful tool to examine the evidence base used in decision‐making. Further study of evidence types used in policymaking processes can help inform research translation and advocacy efforts to produce healthier alcohol policies.

**Key Points**

- Thirty‐one different evidence types were identified in an alcohol policy process.
- While the participating public preferenced studied knowledge, policymakers preferenced practice knowledge.
- We propose an inductive classification framework for mapping the evidence types used in alcohol policymaking processes.

## 1 INTRODUCTION

Alcohol policies shape the alcohol‐induced harms experienced by communities [1] by determining alcohol availability [2], incentivising consumption patterns [3] and ensuring access to treatment [4]. Although alcohol and alcohol‐related harms have been studied extensively [5], the evidence base that is used in alcohol policymaking is not well understood. Gaining a better understanding of what informs alcohol policymaking is vital to reshaping these policies [6] and, with them, community experiences' of alcohol and alcohol‐related harm.

Research on alcohol policymaking has taken up O'Brien's [7] call to map the alcohol industry's influence on policymaking [7, 8, 9, 10, 11, 12, 13, 14], to the near exclusion of all other policy actors and facets; notwithstanding Fitzgerald's notable exception that documented public health policy actors' frustration with policymakers' decision making [15]. Given the comparisons researchers draw between alcohol regulatory policy and tobacco regulatory policy [13, 16] this outsized attention to industry efforts may be warranted. However, consequentially, little is known about evidence use in alcohol policy outside of the alcohol industry's tenuous relationship with evidence [7]. We know that a wide array of knowledges and evidence are introduced in policymaking processes in public health [17], and that certain knowledges are privileged by policymakers [18]. At the local [19] and individual licence [20] level of alcohol policymaking researchers have found that policymakers prefer to base their decisions on local knowledge [21]. This is in‐line with public health studies that found policymakers prefer using local data [22, 23] in their decision‐making processes. It is unclear whether policymakers rely on a similar set of evidence in alcohol policymaking at the state level and when that evidence is used by different policy actors. This study addresses this gap by cataloguing the different types of evidence used within a case study of alcohol policymaking and mapping when those different types of evidence were taken up by policy actors at different phases of the process. In describing and mapping the multiple types of evidence, knowledge and information, we use the generic term ‘evidence’ to mean any and all information supplied in support of a claim, position or argument [24, 25, 26].

The aims of this study were to:

1. Develop a pilot classification scheme for identifying the types of evidence used in alcohol policymaking.
2. Identify the types of evidence that were favoured by different policy actors at different phases in a case study of alcohol policymaking.

### 1.1 The case study: The Joint Select Committee for Sydney's night‐time economy

New South Wales (NSW) state government introduced legislative action (in 2014) in response to two highly publicised cases of non‐domestic assault in which the victims died (in 2012–2013). Both victims were young men who were assaulted by strangers while walking down the streets of Kings Cross (a popular entertainment strip in a central residential area neighbouring the central business district) between 9:00 and 10:00 PM [27, 28]. The assaults were portrayed in the media as emblematic of an epidemic of alcohol‐fuelled violence striking Sydney [29, 30, 31]. The NSW government's legislative package to address alcohol‐related violence was announced in February 2014; these measures included restrictions on glassware, temporal restrictions on services of certain types of beverages, restricted entries and re‐entries to all venues in area post 1:30 AM (the lockout), mandatory ID scanners in select venues and last drinks at 3:00 AM. They affected two late‐night entertainment precincts: the central business district and Kings Cross [32]. Two large gambling operations, The Star Casino and the anticipated Crown Casino (which was in development at the time) were among the few centrally located venues that were exempt from the legislation [33]. The policies were designed to reduce alcohol‐related violence, although the mechanism through which they proposed to do so is unclear [34, 35, 36]. The measures were evaluated by the NSW Bureau of Crime Statistics and Research [37, 38, 39, 40, 41] multiple times over the course of 5 years; by emergency medicine clinicians [42, 43, 44, 45] who evaluated 12–48 months of data; and by independent researchers [46, 47] who evaluated effects after 12 months and 5 years. In total, 11 studies were conducted by researchers, as well as an independent statutory review conducted 2 years post implementation [48] as mandated by the legislation. All the evaluations found the policy to be effective and cited overall reductions in non‐domestic violence assaults [37, 40, 41, 42, 43, 44, 45, 47, 49, 50]. These evaluations relied predominantly on administrative data (crime and hospital data), as is common in alcohol policy evaluations focusing on harms. Two studies incorporated other types of data: the state treasury department's analysis contained a cost–benefit analysis as well as surveys of alcohol‐related businesses [51]; and Hughes and Weedon‐Newstead conducted focus groups with residents of affected entertainment areas [46]. The laws were heralded as a public health success story in the media [52], yet remained deeply unpopular with many among the city's youth [53], alcohol industry [54] and much of its business sector [55]. Advocacy to repeal the laws included multiple mass protests and rallies [56], petitions and political organising which included the foundation of a political party (Keep Sydney Open) [57].

**FIGURE 1:**
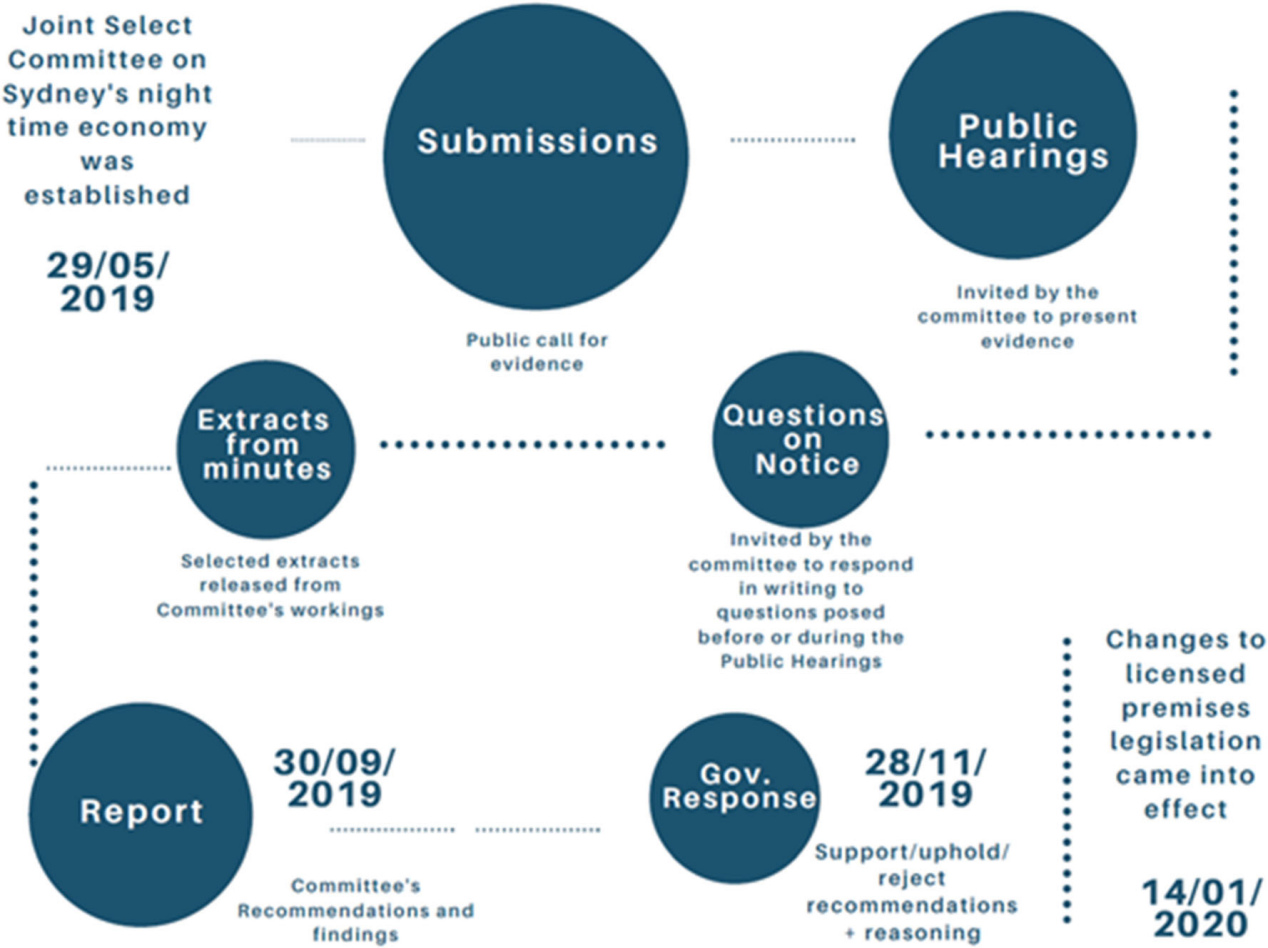
The process of the Joint Select Committee on Sydney's night‐time economy flow chart

The Joint Select Committee on Sydney's Night Time Economy (henceforth the Committee) was established in May 2019 to conduct an inquiry into whether there was a proper balance between community safety and a vibrant night‐time economy [58]; by way of a vote carried in both houses of parliament. Members were selected from the government and opposition in both the upper and lower houses for a fixed term. Committee staff were appointed to assist in, and organise the proceedings and the report [59]. The committee solicited evidence from the public at large in the form of submissions by publishing the terms of reference of the Committee on the government website and in the media. The Committee called for submissions that discussed: (i) maintaining and enhancing community safety; (ii) maintaining and enhancing individual and community health outcomes; (iii) ensuring existing regulatory arrangements in relation to individuals, businesses and other stakeholders, including Sydney's lockout laws, remain appropriately balanced; (iv) enhancing Sydney's night‐time economy; and (v) any other directly relevant matters. The Committee collected submissions, conducted 3 days of public hearings, independent observations and produced a summary report along with recommendations titled: Sydney's night‐time economy (see Figure 1). The state government was required (by way of legislation) to respond to the Committee's recommendations.

## 2 METHODS

### 2.1 Identifying the types of evidence used in the case study

The body of work consists of policy documents collated from the inquiry's six phases (Table 1). All data are in the public domain [60]. Five of the sets of documents were downloaded on 27 February 2020, and the sixth set of documents (responses to questions on notice) was downloaded on 30 April 2021.

**TABLE 1:** Body of work used in analysis of Joint Select Committee on Sydney's night‐time economy

Dataset	Policy phase	Important dates	No. of participants/meetings/reports	Pages
Submissions	1	Open 4/06/2019–2/07/2019	793 received, 282 published	2065 pages published, inc. 282 title pages
Transcripts from public hearings	2	Held on 5/08, 9/08, 12/08/2019	79 witnesses	226 pages published
Responses to questions on notice	3	5/08/2019–30/09/2019	42 received	274 pages published, inc. 42 title pages
Extractions from meetings minutes	4	Held on 4/06, 9/06, 23/07, 5/08, 9/08, 12/08, 26/09/2019	Extracted minutes from 7 meetings published	39 pages published (appendix 6 of report)
Committee report	5	Tabled 30 September 2019	1	93 pages (Report + appendix 1–3)
Government response	6	Released 28 November 2019	1	7 pages
Total				2704 pages

The Committee published 282 submissions out of the 793 received submissions. Submissions were excluded from publication if: (i) the author requested that the submission not be made public; (ii) at the Committee's discretion, the submission was authored by an individual and less than 250 words in length (see appendix six in the Committee's report [59]). Only published submissions were included in the body of work.

To map the types of evidence used in this case study we conducted an iterative content analysis [61] of the corpus of documents using NVivo12 pro. We drafted an initial codebook to classify the different types of evidence (see Table S4). This codebook drew on prior research on evidence and knowledge in the study of evidence based policy [25, 62, 63], evidence based practice [26, 64], evidence utilisation [65, 66] and public opinion [67]. The first round of coding was deductive, coding the dataset in its entirety according to the codebook. We used two questions to guide the application of the codebook: (i) is this information or knowledge being used as evidence in this context (i.e., is this information evidence according to our definition?); (ii) which of the eight types of knowledge identified in the codebook is most suitable here. Codes were then refined inductively during three iterative rounds of recoding. In these rounds we reviewed the data that had been coded against each type individually and applied the following two questions: (i) how did this information come to be known (e.g., was it collected—and if so how, or was it learned through doing, or living in a certain area etc.?); and (ii) what is the source of this information (e.g., which type of research data was used, or which type of practice or profession). We used the responses to these questions to create a refined codebook of 31 discrete codes that represented the types of evidence used in the case study. One hundred and six pages of all data were selected at random and submitted to an external researcher along with the refined codebook for cross‐coding (this represents twice the amount of data recommended to assess intercoder reliability [68]). The median agreement score across all datasets was 82.73% (see Table S1), which is considered acceptable [68, 69]. Once the evidence types were constructed, we grouped the types into four larger classes of evidence. The groupings were based on the source of the information the type of evidence drew on.

### 2.2 Identifying the types of evidence that were favoured by different policy actors at different phases

We collected the counts of references to each code across the six phases of the dataset, to see how many data units were coded against each evidence type at each phase. These were then transferred into Excel to calculate how often each type of evidence was referenced or cited in a policy phase. Here, these acts of reference and citation are understood as representations of use of evidence in the studied process. We identified citations in the submissions phase as reflective of the evidentiary preferences of the participating public. Citations in the hearings and questions on notice phases represented a mix of the preferences of the participating public, as well as a set of policymakers, as they were curated by the latter. Citations in the meetings phase were not used to analyse preferences because this particular phase of the policy process was not eliciting or presenting forms of evidence, but rather debating the evidence (knowledge types) that had already been presented to the committee. Finally, we identified citations in the report and response phases as reflective of the evidentiary preferences of policymakers.

## 3 RESULTS

### 3.1 Types of evidence used in the case study

Using content analysis, we classified 5956 separate evidence referrals and citations into 31 types of evidence. These are presented in Table 2, see Table S2 for more detail. As can be seen, 31 differentiated evidence types were identified. These types represent the wide array of information the participating public and two subsequent groups of policymakers cited as evidence to support their claims, recommendations, and policy positions, and ranged from lived experience through to various kinds of scientific research. In relation to the latter, several different types of quantitative research data (as detailed in Table 2) were cited, alongside qualitative research and references to scientific consensus. Beyond the expected categories of ‘lived experience’ and scientific research, there was also evidence of public opinion, speculation, legal knowledge, practice knowledge and a variety of other evidence types that have rarely been documented in policy processes. The extent to which each of the 31 types of evidence appeared at each of the six phases of the policy process varied.

**TABLE 2:** Types of evidence cited in the Joint Select Committee on Sydney's night‐time economy process

Class	Evidence type	Definition
Person knowledge	Historical background	A description of (pertinent) preceding events.
	Lived experience	The personal and unique experiences of people [70].
	Local knowledge	Information pertaining to, produced by and within the confines of a local community and its immediate environment. This information is not necessarily accepted by all or many in a community as reliable [71].
	Observation	Refers specifically to information gathered directly by the Committee members during in‐person on‐site visits they conducted as part of their committee work.
	Quoting people of note	Quotations from text or speech of people of note (i.e., politicians and famous people).
	Speculation	Inferences derived from questioning policies and motivations, often consequentialist in thinking.
Practice knowledge	Alcohol industry	Knowledge of the practitioners working in the alcohol industry as well as those of non‐practitioners, so long as it pertains to the practice rather than the person.
	Business interests	Knowledge of the practitioners working in the business sector (excluding alcohol) as well as those of non‐practitioners, so long as it pertains to the practice rather than the person.
	Data science	Knowledge of the practitioners working with scientific, or otherwise specialised data and analysis, as well as those of non‐practitioners, so long as it pertains to the practice rather than the person. Does not include findings.
	Entertainment industry	Knowledge of the practitioners working (paid and unpaid) in the entertainment industry as well as those of non‐practitioners, so long as it pertains to the practice rather than the person. Does not include licensed premises or hospitality.
	Governance	Knowledge of the practitioners working in government (local as well as state), as well as those of non‐practitioners, so long as it pertains to the practice rather than the person.
	Health	Knowledge of the practitioners working in health as well as those of non‐practitioners, so long as it pertains to the practice rather than the person. Does not include scientific or statistical information.
	Law enforcement	Knowledge of the practitioners working in law enforcement as well as those of non‐practitioners, so long as it pertains to the practice rather than the person.
	Tourism	Knowledge of the practitioners working in tourism as well as those of non‐practitioners, so long as it pertains to the practice rather than the person.
	Transport	Knowledge of the practitioners working in transport as well as those of non‐practitioners, so long as it pertains to the practice rather than the person.
Shared knowledge	Analogical reasoning	Reliance on a comparison between two objects or situations, while highlighting the ways in which they are similar [72].
	Common knowledge	Information that is likely to be accepted as reliable at face value by everyone, or almost everyone, in a community [73].
	Law	References to legislation and regulations across local, state and federal levels.
	Public opinion (qualitative)	Views presented as believed to be prevalent among the public.
	Public opinion (quantitative)	Information gained from public, professional or market research polls. Does not include scientific surveys.
	Policies, plans and strategies	Strategic thinking (formal and semi‐formal) including principles, guidelines, strategies or plans to achieve organisational objectives [74].
Studied knowledge	Case study	A documented example pertaining to the central problem, which either illustrates the problem or the strategies to resolve it, or both [75].
	Qualitative research	Qualitative research findings or data. Qualitative is understood broadly here as non‐numeric.
	Quantitative: Consumption data	Quantitative research findings or data pertaining to the consumption of alcohol and other drugs. Quantitative is understood broadly here as numeric.
	Quantitative: Crime data	Quantitative research findings or data pertaining to criminal activity. Quantitative is understood broadly here as numeric.
	Quantitative: Economic data	Quantitative research findings or data pertaining to economic activity (including foot traffic). Quantitative is understood broadly here as numeric.
	Quantitative: Emergency department data	Quantitative research findings or data pertaining to hospital emergency departments. Quantitative is understood broadly here as numeric.
	Quantitative: Health data	Quantitative research findings or data pertaining to public health. Quantitative is understood broadly here as numeric.
	Quantitative: Risk of violence	Quantitative research findings or data pertaining to the risk of violence (alcohol related). Quantitative is understood broadly here as numeric.
	Scientific consensus	Evidence that appealed to a near consensus, or particularly strong evidence bases.
	Statutory reviews, committees, commissions and inquiries	Evaluations of acts, policies or policy issues initiated by government actors. Can be conducted by appointees, members of parliament, elected officials or staff.

The 31 types of evidence can be organised into four classes of evidence (see Table 2): person knowledge, shared knowledge, practice knowledge and studied knowledge, based on the source of the evidence. Person knowledge represents types of knowledge which are derived from personal experience. The locus of the knowledge is located within the individual. Shared knowledge refers to generalised knowledge that is located within a public sphere. For this category of shared knowledge, the locus of the knowledge is organised within a group or a public. Borrowed from the study of medicine, practice knowledge is also known as professional knowledge [76]. Practice knowledge encompasses the types of knowledge derived from the continued practice of a professional, semi‐professional, or personal endeavour. These include the kinds of knowledge gained through interactions with peers, mentors and clients, experience, professional guides and a priori knowledge [77]. Here, the knowledge and experiences of both practitioners and non‐practitioners were included, so long as it focused on the practice itself. Studied knowledge represents the types of knowledge that were purposefully collected or collated to be informative on a subject.

### 3.2 Mapping policy actors' evidentiary preferences

**FIGURE 2:**
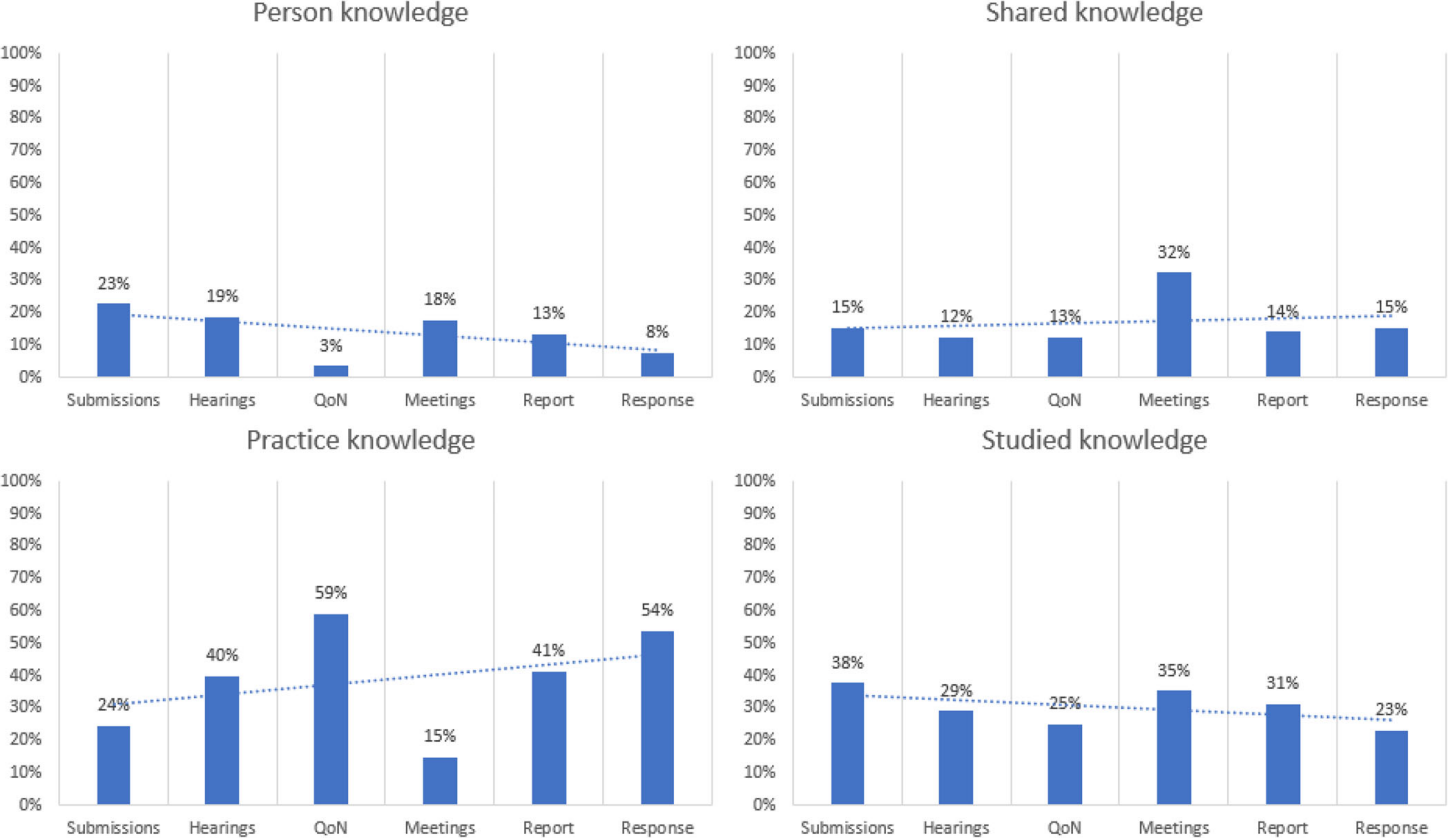
Evidence utilisation by phase in the Joint select committee on Sydney's night‐time economy 2019

Descriptive citation rates were calculated in Excel (see Figure 2 and Table S3). The distribution of citations was heavily concentrated in the submissions phase, as measured through both instances of citations (59%) and range of types (30 out of 31). Therefore, total evidence utilisation rates are heavily skewed by the evidence utilisation rates cited in the submissions phase.

Thirty of the 31 evidence types were cited in the submissions phase (*n* = 30) and in the hearings phase (*n* = 30); fewer were used in the Questions on notice phase (*n* = 24). A smaller number appeared in the later phases of the policy process, with Committee members referring to 14 of the different evidence types in the meeting phase, and then 26 evidence types in the report phase. For the final phase, the NSW government response to the findings, seven evidence types were cited.

Studied knowledge and practice knowledge are cited evenly throughout the policy process (34% and 33% respectively, see Table S3). The participating public preferenced studied knowledge, as during the submissions phase, studied knowledge was the largest category of citations. Policymakers preferenced practice knowledge, as practice knowledge accounted for most of the evidence base throughout the rest of the phases (40% in the hearings, 59% of questions on notice, 41% of the report and 54% of the response) except for during the meetings phase. In terms of evidence utilisation, the meetings are an outlier, with studied knowledge and shared knowledge citations (35% and 32%) nearly double the amount of citations of person knowledge and practice knowledge (18% and 15%). As this phase was reflective of debates regarding findings, it suggests that studied knowledge and shared knowledge were subject to more debate than person or practice knowledge. These results indicate that the application of a classification framework for evidence together with calculations of citations can produce insights regarding the different evidence types used in policymaking, when they are used and by whom.

## 4 DISCUSSION

Previous research on alcohol policymaking has focused on the alcohol industry's weaponization of evidence to exert influence on policymaking [14]. However, a narrow view of the kinds of knowledge that constitute evidence [78, 79, 80] and policy actors obscures a nuanced understanding of the evidence environment, the alcohol industry's influence and policymaking processes. The classification framework piloted here adds much‐needed context to those endeavours by mapping the other evidence types that are also used in the field.

This classification framework corresponds with models of knowledge and evidence used in medical practice [64], social work [65] and policymaking [22], as detailed in Table 3. Comparatively, our proposed classification framework catalogues a far broader set of evidence types than previously studied. The closest model to our framework is the medical model [64], as it includes similar classes of knowledge to our proposed framing. Here the main point of difference is that our framework was developed to map evidence use in a multi‐actor field, while the medical model maps evidence in a binary field (patient/clinician). Both the medical model [64] and the social work model [65] used a system to classify different types of knowledge; the medical model used the source of knowledge as the point of difference and the social work model used the status of knowledge as the point of difference. The policymaking model [22] does not discuss how their types were assembled. Our classification framework contributes to these by covering a wider range of types, and a proposed tool through which to define evidence, and then differentiate between types.

**TABLE 3:** Comparing models of evidence across policymaking, social work and clinical practice

Framework or model	Kowalski et al.	Rycroft‐Malone et al. [64]	Livingston [65]	Oliver and de Vocht [22]
*N* = evidence types	31	4	15	11
Organising categories or types				
	Personal knowledge	Patient experience	Non‐codified knowledge. Personal knowledge	Local data
	Practice knowledge	Clinical experience	Non‐codified knowledge	Practice guidelines
	Shared knowledge	Information from the local context		
	Studied knowledge	Research	Codified knowledge	Joint needs assessments. Experimental or trial data. Qualitative research studies. Public health surveillance data. Health impact assessments. Survey/questionnaire data. Meta‐analyses. Systematic review. Other

By applying our proposed framework, we found that policymakers favoured practice knowledge (see Figure 2), particularly alcohol industry practice knowledge (see Table S3), over all other types of available evidence in the process, in the report and response phases. This extensive utilisation is both surprising and yet somewhat in‐line with current thinking in the study of evidence utilisation [22] and alcohol policy [8, 9, 11, 78, 80, 81]. The class of practice knowledge relates to both tacit knowledge and organisation knowledge in the field [22] and industry‐centred knowledge. As such this finding is consistent with studies that found policymakers favour knowledge that is derived from operational or practice activities [25]. Furthermore, our findings confirmed that the alcohol industry is an influential policy actor [8, 9, 11, 78, 80, 81], as the industry produced evidence that was favoured by policymakers.

This study has a few limitations. First, the vast majority of received submissions were not made public by the Committee. Almost all the unpublished submissions were authored by individuals, meaning the participating public represented here is incomplete. The published submissions represent submissions authored by organisations to a large extent. Hence, the finding relating to the participating public's reliance on studied knowledge should be interpreted accordingly, that is, participating organisations likely value studied knowledge, and rely on a broad range of evidence types to support their claims and positions. It is not possible to determine which types of evidence and knowledges would be found in the unpublished submissions. Future research centring individuals' and publics of individuals' participation in policy [82] and usage of evidence is warranted to study publics' relationship with evidence in policymaking.

Second, this study used a contextual approach to define evidence, that is, information was coded as evidence because it was used by a speaker to support a claim, recommendation or position. Different conceptualisations of evidence would have led to different coding structures and proposed frameworks. As our framework was constructed through an iterative coding methodology, there may be a small degree of overlap between different categories (depending on the degree of nuance applied by each coder). In its current iteration, our proposed framework reflects the policy process it was derived from rather than representing an exhaustive framework with completely mutually exclusive categories.

Third, this study used a case study dealing with alcohol policy specifically in the night‐time economy. This may affect the reproducibility of some of the evidence types represented here in other facets of alcohol policy, such as taxation and marketing. Future applications of the framework to other facets of alcohol policy are warranted to map the breadth of evidence types used in alcohol policy. Nevertheless, this pilot study proposes a framework through which to draw out different types of evidence and clearly establishes that a wide range of evidence types were used in alcohol policymaking by the participating public and policymakers.

In summary, in this study we proposed a classification framework for the types of evidence used in alcohol policymaking processes. We applied the framework to a case study of deliberative policymaking and found that practice knowledge was favoured over studied knowledge by policymakers. We grouped the 31 evidence types into four classes of evidence for practical reasons. Grouping the types into four classes of evidence simplified the framework and increased the possible applications of the framework both within this dataset, and (hopefully) as a tool with which to analyse other datasets. Possible applications range from applying the current framework to other processes of alcohol policymaking, to potential future adaptations of the framework to other fields of policymaking using the same classification process. This approach represents a simple tool to study evidence types and their utilisation in policymaking.

## AUTHOR CONTRIBUTIONS

Each author certifies that their contribution to this work meets the standards of the International Committee of Medical Journal Editors.

## FUNDING INFORMATION

Claire Wilkinson is supported by an NHMRC Early Career Fellowship (11402942). Michael Livingston is funded via an ARC Future Fellowship (FT210100656). Alison Ritter is funded via an NHMRC Senior Research Fellowship (APP1136944). Michala Kowalski is supported by a UNSW Scientia PhD scholarship.

## CONFLICT OF INTEREST

None to declare.

## Supporting information


**Table S1.** Intercoder reliability results.
**Table S2.** Typology of evidence cited in the Joint Select Committee on Sydney's night time economy process
**Table S3.** Citation dataset
**Table S4.** Initial codebook

## References

[1] Casswell S , Huckle T , Wall M , Parker K , Chaiyasong S , Parry CDH , et al. Policy‐relevant behaviours predict heavier drinking and mediate the relationship with age, gender and education status: analysis from the International Alcohol Control Study. Drug Alcohol Rev. 2018;37:S86–95. 29464804 10.1111/dar.12669 PMC6635757

[2] Gray‐Phillip G , Huckle T , Callinan S , Parry CDH , Chaiyasong S , Cuong PV , et al. Availability of alcohol: location, time and ease of purchase in high‐ and middle‐income countries: data from the International Alcohol Control Study. Drug Alcohol Rev. 2018;37:S36–44. 29582496 10.1111/dar.12693 PMC6120539

[3] Devilly GJ , Greber M , Brown K , Allen C . Drinking to go out or going out to drink? A longitudinal study of alcohol in night‐time entertainment districts. Drug Alcohol Depend. 2019;205:107603. 31605959 10.1016/j.drugalcdep.2019.107603

[4] Chalmers J , Ritter A , Berends L , Lancaster K . Following the money: mapping the sources and funding flows of alcohol and other drug treatment in Australia. Drug Alcohol Rev. 2016;35:255–62. 26424113 10.1111/dar.12337

[5] Babor T , Caetano R , Casswell S , Edwards G , Giesbrecht N , Graham K , et al. Alcohol: no ordinary commodity: research and public policy. New York: Oxford University Press; 2010. p. 280–3.

[6] Oliver K , Lorenc T , Innvær S . New directions in evidence‐based policy research: a critical analysis of the literature. Health Res Policy Syst. 2014;12:34. 25023520 10.1186/1478-4505-12-34 PMC4107868

[7] O'Brien KS , Carr SM . Commentary on de Bruijn et al. (2016): Effective alcohol marketing policymaking requires more than evidence on alcohol marketing effects—research on vested interest effects is needed. Addiction. 2016(111):1784–5. 10.1111/add.13489 27605080

[8] Avery MR , Droste N , Giorgi C , Ferguson A , Martino F , Coomber K , et al. Mechanisms of influence: alcohol industry submissions to the inquiry into fetal alcohol spectrum disorders. Drug Alcohol Rev. 2016;35:665–72. 27246440 10.1111/dar.12399

[9] Miller M , Wilkinson C , Room R , O'Brien P , Townsend B , Schram A , et al. Industry submissions on alcohol in the context of Australia's trade and investment agreements: a content and thematic analysis of publicly available documents. Drug Alcohol Rev. 2021;40:22–30. 33230913 10.1111/dar.13219

[10] Cullen D , Smith K , Collin J . ‘Half‐cut’ science: a qualitative examination of alcohol industry actors' use of peer‐reviewed evidence in policy submissions on minimum unit pricing. Evid Policy. 2019;15:49–66.

[11] Stafford J , Kypri K , Pettigrew S . Industry actor use of research evidence: critical analysis of Australian alcohol policy submissions. J Stud Alcohol Drugs. 2020;81:710–8. 33308398

[12] McCambridge J , Kypri K , Sheldon TA , Madden M , Babor TF . Advancing public health policy making through research on the political strategies of alcohol industry actors. J Public Health (Oxf). 2020;42:262–9. 31220307 10.1093/pubmed/fdz031 PMC7297281

[13] McCambridge J , Mialon M , Hawkins B . Alcohol industry involvement in policymaking: a systematic review. Addiction. 2018;113:1571–84. 29542202 10.1111/add.14216 PMC6100095

[14] Bartlett A , McCambridge J . Doing violence to evidence on violence? How the alcohol industry created doubt in order to influence policy. Drug Alcohol Rev. 2022;41:144–52. 34288169 10.1111/dar.13354

[15] Fitzgerald N , Egan M , De Vocht F , Angus C , Nicholls J , Shortt N , et al. Exploring the impact of public health teams on alcohol premises licensing in England and Scotland (ExILEnS): Procotol for a mixed methods natural experiment evaluation 11 medical and health sciences 1117 public health and health services. BMC Med Res Methodol. 2018;18:123. 30400776 10.1186/s12874-018-0573-z PMC6219046

[16] Kypri K , McCambridge J , Robertson N , Martino F , Daube M , Adams P , et al. ‘If someone donates $1000, they support you. If they donate $100 000, they have bought you’. Mixed methods study of tobacco, alcohol and gambling industry donations to Australian political parties. Drug Alcohol Rev. 2019;38:226–33. 30474155 10.1111/dar.12878

[17] Dobrow MJ , Goel V , Upshur REG . Evidence‐based health policy: context and utilisation. Soc Sci Med. 2004;58:207–17. 14572932 10.1016/s0277-9536(03)00166-7

[18] Ritter A . The privileged role of researchers in ‘evidence‐based’ policy: implications and engagement of other voices. Drugs Alcohol Today. 2015;15:181–91.

[19] Martineau FP , Graff H , Mitchell C , Lock K . Responsibility without legal authority? Tackling alcohol‐related health harms through licensing and planning policy in local government. J Public Health (Oxf). 2014;36:435–42. 23933915 10.1093/pubmed/fdt079 PMC4181422

[20] Wilkinson C . Local input in issuing liquor licenses: contemporary policy issues in historical context. Melbourne: University of Melbourne; 2017.

[21] Phillips G , Green J . Working for the public health: politics, localism and epistemologies of practice. Sociol Health Illn. 2015;37:491–505. 25682916 10.1111/1467-9566.12214

[22] Oliver KA , de Vocht F . Defining ‘evidence’ in public health: a survey of policymakers' uses and preferences. Eur J Pub Health. 2015;27(Suppl 2):112–7. 10.1093/eurpub/ckv082 26163467

[23] Whitehead M , Petticrew M , Graham H , Macintyre SJ , Bambra C , Egan M . Evidence for public health policy on inequalities: 2: assembling the evidence jigsaw. J Epidemiol Community Health. 2004;58:817–21. 15365105 10.1136/jech.2003.015297 PMC1763350

[24] Becker HS . Evidence. Chicago, London: University of Chicago Press; 2017.

[25] Sedlačko M , Staronova K . An overview of discourses on knowledge in policy: thinking knowledge, policy and conflict together. Cent Eur J Public Policy. 2015;9:10–53.

[26] Greenhalgh T . Intuition and evidence‐‐uneasy bedfellows? Br J Gen Pract. 2002;52:395–400. 12014539 PMC1314297

[27] Olding R , Davies L , Ralston N . Teen accused of killing Thomas Kelly went on crime spree: police. The Sydney Morning Herald 19/07/2012.

[28] Needham K , Smith A . Daniel Christie dies following king‐hit punch. The Sydney Morning Herlad 11/01/2014.

[29] Tomsen S. Blame it on the booze: mass drinking drives Sydney's violence. The Conversation 24/07/2012.

[30] Pilgrim J. Stop alcohol‐fuelled violence ‐ it's killing our youth. The Sydney Morning Herald. 03/12/2013.

[31] Needham K , Smith A . Daniel Christie latest fatility in epidemic of street violence. The Sydney Morning Herald. 12/01/2014.

[32] Lockout to commence from 24 February [press release]. NSW Government, 5/02/2014.

[33] Koziol M. And the winner of Sydney's lockout laws is… Star casino! The Sydney Morning Herald. 29/08/2014.

[34] Arditi J . Liquor, licenses and lockouts. Sydney: NSW Parliamentary Library Research Service; 2008.

[35] Graham K , Homel R . Raising the bar. New York: Taylor& Franis; 2011.

[36] Miller P , Curtis A , Chikritzhs T , Toumbourou J . Interventions for reducing alcohol supply, alcohol demand and alcohol‐related harm: final Report 2015.

[37] Menéndez P , Weatherburn D , Kypri K , Fitzgerald J . Lockouts and last drinks: the impact of the January 2014 liquor licence reforms on assaults in NSW, Australia. Crime Justice Bulletin. 2015;183:1–12.

[38] Donnelly N , Weatherburn D , Routledge K , Ramsey S , Mahoney N . Did the ‘lockout law’ reforms increase assaults at the Star casino, Pyrmont? Star. 2016;1(2).

[39] Donnelly N , Poynton S , Weatherburn D . Effect of lockout and last drinks laws on non‐domestic assaults in Sydney: an update to September 2016, the BOCSAR NSW Crime and Justice Bulletins 2017:12.

[40] Menéndez P , Kypri K , Weatherburn D . The effect of liquor licensing restrictions on assault: a quasi‐experimental study in Sydney, Australia. Addiction. 2017;112:261–8. 27658620 10.1111/add.13621

[41] Donnelly N , Poynton S . The effect of lockout and last drinks laws on non‐domestic assaults in Sydney: an update to march 2019. Crime Justice Bulletin. 2019;142:142.

[42] Fulde GW , Smith M , Forster SL . Presentations with alcohol‐related serious injury to a major Sydney trauma hospital after 2014 changes to liquor laws. Med J Aust. 2015;203:366. 26510806 10.5694/mja15.00637

[43] Dinh MM , Wu J , Ivers R . Has there been a shift in alcohol‐related violence to neighbouring inner city ‘lockout law’ exclusion areas in Sydney? Emerg Med Australas. 2016;28:611–3. 10.1111/1742-6723.12645 27357328

[44] Chopra S , van der Rijt RG , Ngo Q , Clarke FK , Southwell‐Keely JP , Robledo K , et al. A comparison of maxillofacial trauma before and after implementation of lockout laws in Sydney. Australas J Plast Surg. 2018;1:64–70.

[45] Holmes RF , Lung T , Fulde GW , Fraser CL . Fewer orbital fractures treated at St Vincent's Hospital after lockout laws introduced in Sydney. Med J Aust. 2018;208:174. 29490212 10.5694/mja17.00564

[46] Hughes CE , Weedon‐Newstead AS . Investigating displacement effects as a result of the Sydney, NSW alcohol lockout legislation. Drugs Educ Prev Policy. 2018;25:386–96.

[47] Kypri K , Livingston M . Incidence of assault in Sydney, Australia, throughout 5 years of alcohol trading hour restrictions: controlled before‐and‐after study. Addiction. 2020;115:2045–54. 32107857 10.1111/add.15025

[48] Callinan IDF . Review of amendments to the liquor act 2007 (NSW). Liquor and Gaming NSW; 2016 13/09/2016.

[49] Donnelly N , Weatherburn D , Routledge K , Ramsey S , Mahoney N . Did the ‘lockout law’ reforms increase assaults at The Star casino, Pyrmont? Bureau Brief. Sydney: State of NSW Department of Justice; 2016; Report No. 114.

[50] Donnelly N , Poynton S , Weatherburn D . The effect of lockout and last drinks laws on nondomestic assaults in Sydney an update to September 2016. Sydney, NSW: NSW Bureau of Crime Statistics and Research, justice do; 2017. Report No. 201.

[51] The Treasury . Evaluation of the Sydney CBD entertainment precinct plan of management. New South Wales: NSW Government; 2016.

[52] Fitzgerald R. Why all Sydneysiders should be grateful for the lockout. The Sydney Morning Herald 20/03/2016.

[53] Hunt E. ‘They're treating us like children’: a generation rages against Sydney's lockout laws. The Guardian 27/02/2016.

[54] Kings cross businesses seek compensation for impact of lockout laws. The Sydney Morning Herald. 7/08/2015.

[55] Yun J. $16 billion: That's how much Sydney's lock‐out laws have cost the city's economy. Yahoo!finance 12/02/2019.

[56] NEWS A . Keep Sydney open: thousands attend protest against lockout laws, Jimmy Barnes backs campaign. ABC News 9/10/2016.

[57] Visentin L. Keep Sydney open says election failure has boosted momentum for lockout law repeal. The Sydney Morning Herald. 22/04/2019.

[58] Committee seeking input on Sydney's lockout laws [press release] Parliament house: Parliament of New South Wales 2019.

[59] Joint Select Committee on Sydney's Night Time Economy . Sydney's night time economy. NSW: Parliament of NSW; 2019.

[60] Joint Select Committee on Sydney's Night Time Economy NSW parliament: parliament of New South Wales. New South Wales: Parliament of New South Wales; 2019. Available from: https://www.parliament.nsw.gov.au/committees/listofcommittees/Pages/committee‐details.aspx?pk=260#tab‐resolution

[61] Hsieh H‐F , Shannon SE . Three approaches to qualitative content analysis. Qual Health Res. 2005;15:1277–88. 16204405 10.1177/1049732305276687

[62] Cairney P . The politics of evidence‐based policy making. London: Palgrave Pivot; 2016.

[63] Lancaster K , Treloar C , Ritter A . ‘Naloxone works’: the politics of knowledge in ‘evidence‐based’ drug policy. Health. 2017;21:278–94. 28135864 10.1177/1363459316688520

[64] Rycroft‐Malone J , Seers K , Titchen A , Harvey G , Kitson A , McCormack B . What counts as evidence in evidence‐based practice? J Adv Nurs. 2004;47:81–90. 15186471 10.1111/j.1365-2648.2004.03068.x

[65] Livingston W . Towards a comprehensive typology of knowledge for social work and alcohol. Soc Work Educ. 2014;33:774–87.

[66] Gluckman P . Editor the role of evidence and expertise in policy‐making: the politics and practice of science advice. J Proc R Soc NSW. 2018;151:91–101.

[67] Chinn S , Lane DS , Hart PS . In consensus we trust? Persuasive effects of scientific consensus communication. Public Underst Sci. 2018;27:807–23. 30058947 10.1177/0963662518791094

[68] Lombard M , Snyder‐Duch J , Bracken CC . Practical resources for assessing and reporting intercoder reliability in content analysis research projects. 2010. Available from: http://matthewlombard.com/reliability/index_print.html.

[69] Hallgren KA . Computing inter‐rater reliability for observational data: an overview and tutorial. Tutor Quant Methods Psychol. 2012;8:23–34. 22833776 10.20982/tqmp.08.1.p023 PMC3402032

[70] Given LM . The SAGE encyclopedia of qualitative research methods. Los Angeles, CA: Sage Publications. Inc; 2008.

[71] Nugroho K , Carden F , Antlov H . Local knowledge matters. Power, context and policy making in Indonesia. 1st ed. Bristol: Bristol University Press; 2018.

[72] Bartha P . The Stanford encyclopedia of philosophy (Summer 2022 Edition). In: Zalta EN , editor. Analogy and analogical reasoning. Stanford, CA: Standord University; 2013 Available from: https://plato.stanford.edu/archives/sum2022/entries/reasoning-analogy

[73] MIT . What is common knowledge? MIT; unknown. Available from: https://integrity.mit.edu/handbook/citing-your-sources/what-common-knowledge.

[74] Rosen R . Planning, management, policies and strategies: four fuzzy concepts. Int J Gen Syst. 1974;1:245–52.

[75] Candelaria AL . Protocol for case study writing 2013. Available from: http://politicsandideas.org/protocol-for-case-study-writing/.

[76] Higgs J , Richardson B , Abrandt DM . Developing practice knowledge for health professionals. Edinburgh: Butterworth & Heinemann; 2004.

[77] Estabrooks CA , Rutakumwa W , O'Leary KA , Profetto‐McGrath J , Milner M , Levers MJ , et al. Sources of practice knowledge among nurses. Qual Health Res. 2005;15:460–76. 15761093 10.1177/1049732304273702

[78] McCambridge J , Hawkins B , Holden C . Industry use of evidence to influence alcohol policy: a case study of submissions to the 2008 Scottish government consultation. PLoS Med. 2013;10:e1001431. 23630458 10.1371/journal.pmed.1001431 PMC3635861

[79] McCambridge J , Mialon M . Alcohol industry involvement in science: a systematic review of the perspectives of the alcohol research community. Drug Alcohol Rev. 2018;37:565–79. 29900619 10.1111/dar.12826 PMC6055701

[80] Bertscher A , London L , Orgill M . Unpacking policy formulation and industry influence: the case of the draft control of marketing of alcoholic beverages bill in South Africa. Health Policy Plan. 2018;33:786–800. 29931204 10.1093/heapol/czy049

[81] Martino FP , Miller PG , Coomber K , Hancock L , Kypri K . Analysis of alcohol industry submissions against marketing regulation. PLoS One. 2017;12:e0170366. 28118411 10.1371/journal.pone.0170366 PMC5261775

[82] Lea T . Wild Policy: Indigeneity and the Unruly Logics of Intervention. Stanford, CA: Stanford University Press; 2020.

