# Supplementary material for: Piloting a classification framework for the types of evidence used in alcohol policymaking
Source: Drug Alcohol Rev. 2023 Jan 25;42(3):652–63. doi: 10.1111/dar.13599 (PMC11240880; doi:10.1111/dar.13599)
Supplement: Supplementary file 1 — Table S1. Intercoder reliability results. Table S2. Typology of evidence cited in the Joint Select Committee on Sydney's night time economy process Table S3. Citation dataset Table S4. Initial codebook [file DAR-42-652-s001.docx]

Piloting a classification framework for the types of evidence used in alcohol policymaking supplementary Materials

Table S1: Intercoder reliability results

| Dataset | File size (2% of each dataset) | Kappa Score | Agreement | Disagreement |
| --- | --- | --- | --- | --- |
| Submissions | 67 pages (129637 chars) | 0.6110 | 82.73% | 17.27% |
| Transcripts from Public Hearings | 12 pages (62232 chars) | N/A | 58.82% | 41.17% |
| Responses to Questions on Notice | 13 pages (17183 chars) | 0.4594 | 97.43% | 2.57% |
| Extractions from Meetings minutes | 2 pages (4592 chars) | N/A | 85.71% | 14.28% |
| Committee Report | 5 pages (1013 chars) | N/A | 77.78% | 22.22% |
| Government Response | 7 pages (22534 chars) | 0.5 | 94.29% | 5.71% |
| Median |  | N/A | 82.73% | 17.27% |

Table S2: Typology of Evidence cited in the Joint Select Committee on Sydney’s Night Time Economy process

| Class | Type | Definition | Examples |
| --- | --- | --- | --- |
| Person knowledge | Historical Background | A description of (pertinent) preceding events. | "Oxford Street was the growing antithesis to the toxicity and uniformity of the Cross," - Submission 385, Oxford Art Factory |
|  | Lived Experience | The personal and unique experiences of people.[70] | "This is not the Sydney I grew up in." – Submission 264, Russell White |
|  | Local Knowledge | Information pertaining to, and produced by, both the community and its environment.[71] | "The geography and infrastructure of the Sydney CBD support a diverse night-life” – Sydney’s night time economy -report, pp. vii |
|  | Observation | Refers specifically to information gathered directly by the Committee members during in-person on-site visits they conducted as part of their Committee work. | "The Committee has seen some of that working effectively in Oxford street." - Transcripts from hearings, Monday 5th August 2019, the Hon. Natalie Ward (Chair), pp.13 |
|  | Quoting people of note | Quotations from text or speech of people of note (i.e., politicians and famous people). | “According to the NSW premier, Gladys Berejiklian” – Submission 136, Nicholas Berry |
|  | Speculation | Inferences derived from questioning policies and motivations, often consequentialist in thinking. | "And why? So, the casinos can stay open late?” -Submission 327, Susan Heath |
| Practice knowledge | Alcohol industry | Knowledge of the practitioners working in the alcohol industry as well as those of non-practitioners, so long as it pertains to the practice rather than the person. | “[I]f you have a beverage menu that is more attractive to both sexes, you are probably more likely to have a lower-risk venue.” – Transcripts from hearings, Friday 9th August 2019, testimony of Alec Wagstaff pp.21 |
|  | Business interests | Knowledge of the practitioners working in the business sector (excluding alcohol) as well as those of non-practitioners, so long as it pertains to the practice rather than the person. | "Small business owners, like those operating in the Kings Cross precinct have experienced enormous stress as their livelihoods were threatened by the lockout.” – Submission 383, The Potts Point Partnership |
|  | Data science | Knowledge of the practitioners working with scientific, or otherwise specialized data and analysis, as well as those of non-practitioners, so long as it pertains to the practice rather than the person. Does not include findings. | "Ms CATE FAEHRMANN: Do you think that makes sense from a statistician's point of view? Professor CRIPPS: no, I do not," - Transcripts from hearings, Monday 5th August 2019, testimony of Professor Sally Cripps, pp.29-30 |
|  | Entertainment industry | Knowledge of the practitioners working (paid and unpaid) in the entertainment industry as well as those of non-practitioners, so long as it pertains to the practice rather than the person. Does not include licensed premises or hospitality. | "it is harder to perform (and watch) music now than in any point I can remember”- Submission 249, Daniel Arena |
|  | Governance | Knowledge of the practitioners working in government (local as well as state), as well as those of non-practitioners, so long as it pertains to the practice rather than the person. | "The most effectively managed precincts are those where government and the local community work in partnership” – Submission 780, City of Sydney |
|  | Health | Knowledge of the practitioners working in health as well as those of non-practitioners, so long as it pertains to the practice rather than the person. Does not include scientific or statistical information. | "Our hospital's front-line services were once places of regular violence." - Transcripts from hearings, Friday 9th August 2019, testimony of Dr Paul Thomas Preisz, pp.1-2 |
|  | Law enforcement | Knowledge of the practitioners working in law enforcement as well as those of non-practitioners, so long as it pertains to the practice rather than the person. | "Our resources are also being freed up." - Transcripts from hearings, Friday 9th August 2019, testimony of Pat Gooley pp. 36 |
|  | Tourism | Knowledge of the practitioners working in tourism as well as those of non-practitioners, so long as it pertains to the practice rather than the person. | "[T]here has been a significant price deflation in the market to maintain that occupancy rate." - Transcripts from hearings, Friday 9th August 2019, testimony of Dean Long, pp.65 |
|  | Transport | Knowledge of the practitioners working in transport as well as those of non-practitioners, so long as it pertains to the practice rather than the person. | “[T]he trains stop at 1:30, so it is $100 to get home in a cab” – Submission 1, George Tulloch |
| Shared knowledge | Analogical reasoning | Reliance on a comparison between two objects or situations, while highlighting the ways in which they are similar.[72] | "Imagine closing the economic output of Martin Place every day at lunch time." - Submission 572, Anton Kouzmin |
|  | Common Knowledge | Information that is likely to be accepted as reliable at face value by everyone, or almost everyone, in a community.[73] | "There will ALWAYS be a minority that do the wrong thing." - Submission 069, Trevor Rogers |
|  | Law | References to legislation and regulations across local, state, and federal levels. | "Beyond the State government we have the most complex tax system for alcohol in the world.” - Transcripts from hearings, Friday 9th August 2019, testimony of Alec Wagstaff, pp.18 |
|  | Public Opinion (Qualitative) | Views presented as believed to be prevalent among the public. | "This is not the type of city people want.” – Submission 025, Ryan Diefenbach |
|  | Public Opinion (Quantitative) | Information gained from public, professional or market research polls. Does not include scientific surveys. | "[A] 2019 survey of 34,000 people by Time Out rated Sydney's night life as 48th out of 48." - Submission 657, The Committee for Sydney |
|  | Policies, plans & strategies | Strategic thinking (formal and semi-formal) including principles, guidelines, strategies or plans to achieve organizational objectives.[74] | "On the discussion of the Good Neighbour policy," - Transcripts from hearings, Friday 9th August 2019, the Hon. Natalie Ward (Chair), pp.44 |
| Studied knowledge | Case study | A documented example pertaining to the central problem, which either illustrates the problem or the strategies to resolve it, or both.[75] | "In Berlin Germany, 15,000 tourists come every weekend just for the clubs." - Submission 387, CMAG (Club Music Advisory Group) |
|  | Qualitative Research | Qualitative research findings or data. Qualitative is understood broadly here as non-numeric. | "During our observations in bars in California for the aforementioned NIH-funded study,” – Submission 436, Dr Christopher Morrison |
|  | Quant: consumption data | Quantitative research findings or data pertaining to the consumption of alcohol and other drugs. Quantitative is understood broadly here as numeric. | "Per capita alcohol consumption is at a 50-year low” – Submission 458, Diageo Australia |
|  | Quant: crime data | Quantitative research findings or data pertaining to criminal activity. Quantitative is understood broadly here as numeric. | "Assaults in Kings Cross have decreased by almost 94 per cent between 3:00 am and 6:00 am” – Transcripts from hearings, Friday 9th August 2019, testimony of Pat Gooley, pp. 36 |
|  | Quant: economic data | Quantitative research findings or data pertaining to economic activity (including foot traffic). Quantitative is understood broadly here as numeric. | “[W]e’ve lost thousands of jobs and $16 billion worth of economic activity for Sydney.” – Submission 358, Candy’s Apartment |
|  | Quant: emergency department data | Quantitative research findings or data pertaining to hospital emergency departments. Quantitative is understood broadly here as numeric. | “[E]mergency department presentations at St Vincent’s that are related to acute intoxication have dropped by almost 25%” – Sydney’s night time economy – report, pp. 12 |
|  | Quant: health data | Quantitative research findings or data pertaining to public health. Quantitative is understood broadly here as numeric. | "There is no safe limit of alcohol consumption in relation to cancer risk." - Submission 597, Cancer Council NSW |
|  | Quant: risk of violence | Quantitative research findings or data pertaining to the risk of violence (alcohol related). Quantitative is understood broadly here as numeric. | "Australian research indicates that just 9.78% of alcohol-related injuries occur at licensed venues" - Submission 462, Australian Taxpayers Alliance |
|  | Scientific Consensus | Evidence that appealed to a near consensus, or particularly strong evidence bases. | "[t]he imposition of restrictions on late-night trading is supported by one of the strongest evidence bases in alcohol policy research," - Submission 386, DPMP & CAPR |
|  | Statutory reviews, committees, commissions & inquiries | Evaluations of acts, policies or policy issues initiated by government actors. Can be conducted by appointees, members of parliament, elected officials, or staff. | "As the 2016 Callinan review of the 2014 reforms noted” – Submission 662, Alcohol and Drug Foundation |

Table S3: Citation dataset

| Category | Type of evidence | Submissions | % | Hearing | % | QoN | % | Meeting | % | Report | % | Response | % | Total | Total rates % | |
| --- | --- | --- | --- | --- | --- | --- | --- | --- | --- | --- | --- | --- | --- | --- | --- | --- |
| Person knowledge | Historical background | 50 | 1 | 14 | 1 | 0 | 0 | 0 | 0 | 1 | 0 | 0 | 0 | 65 | 19 | |
|  | Lived Experience | 328 | 9 | 73 | 5 | 0 | 0 | 2 | 6 | 6 | 2 | 0 | 0 | 409 |  |  |
|  | Local Knowledge | 82 | 2 | 127 | 9 | 17 | 2 | 3 | 9 | 23 | 8 | 1 | 8 | 253 |  |  |
|  | Observation | 0 | 0 | 21 | 1 | 0 | 0 | 1 | 3 | 2 | 1 | 0 | 0 | 24 |  |  |
|  | Quotes from people of note | 134 | 4 | 9 | 1 | 3 | 0 | 0 | 0 | 4 | 1 | 0 | 0 | 150 |  |  |
|  | Speculation | 200 | 6 | 28 | 2 | 4 | 1 | 0 | 0 | 2 | 1 | 0 | 0 | 234 |  |  |
| sub total |  |  | 0 |  | 0 |  | 0 |  | 0 |  | 0 |  | 0 | 1135 |  |  |
| Practice knowledge | Alcohol industry | 276 | 8 | 143 | 10 | 64 | 9 | 3 | 9 | 43 | 15 | 4 | 31 | 533 | 33 | |
|  | Business interests | 59 | 2 | 10 | 1 | 2 | 0 | 0 | 0 | 0 | 0 | 0 | 0 | 71 |  |  |
|  | Data science | 32 | 1 | 44 | 3 | 51 | 7 | 0 | 0 | 4 | 1 | 2 | 15 | 133 |  |  |
|  | Entertainment industry | 236 | 7 | 82 | 6 | 30 | 4 | 1 | 3 | 15 | 5 | 0 | 0 | 364 |  |  |
|  | Governance | 70 | 2 | 118 | 8 | 184 | 27 | 0 | 0 | 24 | 8 | 1 | 8 | 397 |  |  |
|  | Health | 65 | 2 | 50 | 3 | 22 | 3 | 0 | 0 | 7 | 2 | 0 | 0 | 144 |  |  |
|  | Law Enforcement | 29 | 1 | 61 | 4 | 20 | 3 | 0 | 0 | 5 | 2 | 0 | 0 | 115 |  |  |
|  | Tourism | 21 | 1 | 28 | 2 | 9 | 1 | 0 | 0 | 1 | 0 | 0 | 0 | 59 |  |  |
|  | Transport | 62 | 2 | 43 | 3 | 25 | 4 | 1 | 3 | 18 | 6 | 0 | 0 | 149 |  |  |
| sub total |  |  | 0 |  | 0 |  | 0 |  | 0 |  | 0 |  | 0 | 1965 |  |  |
| Shared knowledge | Analogical reasoning | 6 | 0 | 7 | 0 | 0 | 0 | 0 | 0 | 0 | 0 | 0 | 0 | 13 | 14 | |
|  | Law | 91 | 3 | 23 | 2 | 19 | 3 | 5 | 15 | 9 | 3 | 2 | 15 | 149 |  |  |
|  | Public opinion qualitative | 154 | 4 | 65 | 4 | 1 | 0 | 0 | 0 | 12 | 4 | 0 | 0 | 232 |  |  |
|  | Public opinion quantitative | 98 | 3 | 19 | 1 | 15 | 2 | 1 | 3 | 6 | 2 | 0 | 0 | 139 |  |  |
|  | Policies, plans & strategies | 82 | 2 | 42 | 3 | 51 | 7 | 5 | 15 | 13 | 5 | 0 | 0 | 193 |  |  |
|  | Common Knowledge | 98 | 3 | 22 | 2 | 0 | 0 | 0 | 0 | 0 | 0 | 0 | 0 | 120 |  |  |
| sub total |  |  | 0 |  | 0 |  | 0 |  | 0 |  | 0 |  | 0 | 846 |  |  |
| Studied knowledge | Case study | 349 | 10 | 181 | 12 | 17 | 2 | 1 | 3 | 26 | 9 | 0 | 0 | 574 | 34 | |
|  | Statutory reviews, committees, commissions & inquiries | 97 | 3 | 20 | 1 | 38 | 6 | 0 | 0 | 4 | 1 | 1 | 8 | 160 |  |  |
|  | Qualitative research | 35 | 1 | 3 | 0 | 0 | 0 | 0 | 0 | 2 | 1 | 0 | 0 | 40 |  |  |
|  | Quant: consumption data | 63 | 2 | 27 | 2 | 23 | 3 | 2 | 6 | 3 | 1 | 0 | 0 | 118 |  |  |
|  | Quant: crime data | 222 | 6 | 64 | 4 | 39 | 6 | 2 | 6 | 25 | 9 | 0 | 0 | 352 |  |  |
|  | Quant: economic data | 310 | 9 | 70 | 5 | 34 | 5 | 6 | 18 | 17 | 6 | 0 | 0 | 437 |  |  |
|  | Quant: emergency department data | 58 | 2 | 12 | 1 | 5 | 1 | 0 | 0 | 3 | 1 | 0 | 0 | 78 |  |  |
|  | Quant: health data | 28 | 1 | 2 | 0 | 7 | 1 | 0 | 0 | 0 | 0 | 0 | 0 | 37 |  |  |
|  | Quant: risk of violence data | 124 | 4 | 43 | 3 | 8 | 1 | 1 | 3 | 8 | 3 | 2 | 15 | 186 |  |  |
|  | Scientific Consensus | 28 | 1 | 0 | 0 | 0 | 0 | 0 | 0 | 0 | 0 | 0 | 0 | 28 |  |  |
| sub total |  |  |  |  |  |  |  |  |  |  |  |  |  | 2010 |  |  |
| Total | | 3487 | 100 | 1451 | 100 | 688 | 100 | 34 | 100 | 283 | 100 | 13 | 100 | 5956 | 100 | |
| Total rates % | | 59 | | 24 | | 12 | | 1 | | 5 | | 0 | |  | |  |

Table S4: initial codebook

| **Anecdote** | **Individual experiences pertaining to the relevant topic** |
| --- | --- |
| **Expert consensus** | **A body of literature** |
| **Practice knowledge** | **Experiences of practitioners of professions concerned with the relevant topic** |
| **Public opinion** | **Public polling results and representations of community interests and wants** |
| **Scientific knowledge (qualitative)** | **Qualitative research in the non-numeric sense** |
| **Scientific knowledge (quantitative)** | **Quantitative research (numeric)** |
| **Speculative knowledge** | **Knowledge regarding possible motivations** |
| **Testimonials** | **Court, hearings, proceedings** |
